# Geographical Distribution of Intestinal Schistosomiasis and Soil-Transmitted Helminthiasis and Preventive Chemotherapy Strategies in Sierra Leone

**DOI:** 10.1371/journal.pntd.0000891

**Published:** 2010-11-23

**Authors:** Joseph B. Koroma, Jen Peterson, Aiah A. Gbakima, Francis E. Nylander, Foday Sahr, Ricardo J. Soares Magalhães, Yaobi Zhang, Mary H. Hodges

**Affiliations:** 1 National Onchocerciasis Control Program, Ministry for Health and Sanitation, Freetown, Sierra Leone; 2 World Health Organization, Freetown, Sierra Leone; 3 Helen Keller International, Freetown, Sierra Leone; 4 University of Sierra Leone, Freetown, Sierra Leone; 5 School of Population Health, University of Queensland, Herston, Queensland, Australia; 6 Regional Office for Africa, Helen Keller International, Dakar, Senegal; London School of Hygiene & Tropical Medicine, United Kingdom

## Abstract

**Background:**

A national baseline mapping of schistosomiasis and soil-transmitted helminthiasis (STH) was performed in Sierra Leone. The aim was to provide necessary tools for the Ministry of Health and Sanitation to plan the intervention strategies in the national integrated control program on neglected tropical diseases according to the World Health Organization (WHO) guidelines for preventative chemotherapy (PCT) and for future monitoring and evaluation.

**Methodology/Principal Findings:**

53 primary schools were randomly selected through a two-staged random sampling throughout the country. Approximately one hundred children aged 5–16 years of age were systematically selected from each school and their stool samples examined in a field laboratory. A total of 5,651 samples were examined. Data were analyzed with multivariable logistic regression models using model-based geostatistics. Spatial analysis predicted that *S. mansoni* infection was positively associated with population density and elevation and that there was a large cluster of high risk of *S. mansoni* infection (prevalence >70%) in the north and most of the eastern areas of the country, in line with the observed prevalence in Kono (63.8–78.3%), Koinadugu (21.6–82.1%), Kailahun (43.5–52.6%), Kenema (6.1–68.9%) and Tonkolili (0–57.3%). Hookworm infection was negatively associated with population density and land surface temperature, and was high across Sierra Leone with a large cluster of high infection risk (prevalence >70%) in the north-eastern part of the country. Remarkably low prevalence of *Ascaris lumbricoides* (7.2%) and *Trichuris trichiura* (3.3%) was recorded when compared with results published in the 1990s.

**Conclusions/Significance:**

Results justify PCT for schistosomiasis for school age children and at-risk adults every year in high-risk communities in five districts and every two years in moderate-risk communities in one more district. The high prevalence of STH, particularly hookworm, coupled with widespread anemia according to a national report in Sierra Leone, suggests all but one district justifying biannual PCT for STH for pre-school children, school age children, and at-risk adults. PCT for STH in the remaining district, Kono is justified annually.

## Author Summary

The common intestinal roundworm, whipworm and hookworm (together known as soil-transmitted helminthes - STHs) together with schistosomes or bilharzia are responsible for extensive ill health, reduced life expectancy and death in sub-Saharan Africa. These diseases are transmitted in areas of poor water supply and sanitation. In order to implement an appropriate national control program, knowledge of the prevalence and geographical distribution of these diseases is required. A national survey was performed in Sierra Leone in 2008. Overall prevalence of intestinal schistosomiasis was 18.4% and that of STHs was 39.1%. Intestinal schistosomiasis was mainly prevalent in the northern and eastern regions while STH is widespread in the country. The results justify routine de-worming for pre-school children, school age children, women of childbearing age, and adults at high risk twice a year. The results also justify using anti-schistosomiasis drug (praziquantel) in school age children, all women of childbearing age, and adults at high risk annually or biennially depending upon the prevalence in the areas.

## Introduction

Schistosomiasis and soil-transmitted helminthiasis (STH), two of the most important neglected tropical diseases, cause serious public health problems in sub-Saharan Africa [1]. There are two main forms of human schistosomiasis in sub-Saharan Africa, intestinal schistosomiasis caused by *Schistosoma mansoni* and urinary schistosomiasis caused by *S. haematobium*. STH is caused by a group of nematodes, most importantly, hookworms, *Ascaris lumbricoides*, and *Trichuris trichiura*. It is estimated that over 200 million people are infected with schistosomes worldwide and about two billion with STHs [2]. These together cause significant morbidity worldwide, with 39 million disability adjusted life years (DALYs) lost each year [3]. With growing global attention on control of the neglected tropical diseases in the last few years, control of these diseases is gathering pace in sub-Saharan Africa with several countries implementing national control programs with financial support from external donors and technical assistance from international organizations [4], [5].

In Sierra Leone, both intestinal and urinary schistosomiasis is known to be prevalent in the northern and eastern regions [6], [7]. STH is endemic throughout the country [6], [8], [9], [10], [11], and the hookworm species present in the country was identified as *Necator americanus* [12]. These diseases, together with the country's other neglected tropical diseases e.g. onchocerciasis and lymphatic filariasis (LF), pose a great threat to the wellbeing of the population, particularly children. Children acquire infections typically from the weaning period as they become mobile and inquisitive, play barefoot and bathe in infected fresh water. Infected children on the marginal level of adequate nutrition will be at risk of growth retardation [13], [14], [15], impaired cognition, and micronutrient deficiencies [16], [17], [18].

Despite the high burden of these diseases in the country, there has never been a national control program on schistosomiasis and STH. However, there has been de-worming activities for STH by non-governmental organizations and the United Nations agencies with albendazole or mebendazole to school age children and pre-school children since 2004 and 2006 respectively. There has also been treatment for lymphatic filariasis added to the existing onchocerciasis control program in six districts bordering Guinea and Liberia since 2007. In 2008, a national integrated NTD Control Program targeting onchocerciasis, LF, schistosomiasis, STH and trachoma was initiated with financial support from the United States Agency for International Development and technical support from Helen Keller International and other partners, using the integrated control strategy according to the preventive chemotherapy (PCT) guidelines recommended by the World Health Organization (WHO) [19].

A national survey of schistosomiasis and STHs was conducted in 2008 to determine the geographical distribution of these diseases in Sierra Leone. We conducted spatial analysis of the disease distribution and provided the disease distribution maps in the country to enable the national integrated NTD control program to plan the integrated control strategy and to determine the disease-specific PCT strategy in each district. The paper presents the prevalence distribution of intestinal schistosomiasis and STH, estimation of at-risk population and corresponding PCT strategy for each district.

## Materials and Methods

### Ethics statement

The national program of NTD control is managed and implemented by the Ministry of Health and Sanitation (MoHS), Sierra Leone. The program involved a national survey on the prevalence of each NTD in order to plan the implementation strategy. The ethical approval for data collection in school children was obtained from the Ethics Committee of the MoHS. Upon arrival at the selected schools, the investigating team met with the community teachers associations in each school, and explained the nature of the survey. Informed consent was verbally given by guardians/parents and recorded by the team leader, as literacy rates are low in Sierra Leone. Once data were collected, the results were anonymized and computerized. No individual identity can be revealed upon publication.

### Country general information

Sierra Leone is in West Africa bordered by Guinea, Liberia and the Atlantic Ocean. It has an area of 71,740 km^2^, a projected population of 5,473,530, a growth rate of 1.8%, a life expectancy of 41 years and an adult literacy rate of 36% in 2008 [20]. The country is administratively divided into 12 districts which are also subdivided into 149 chiefdoms and the Western Area (WA) which is subdivided into 30 zones.

### Sampling

A national cross-sectional survey was conducted in 2008 in order to facilitate the planning of mass drug administration for both schistosomiasis and STH. Although it is known that schistosomiasis is prevalent in Sierra Leone, particularly, in the northern and eastern regions, data were not available in terms of detailed distribution and level of risk of the disease throughout the country. It was decided that all 12 districts including cities plus the Western Area (excluding the capital Freetown) were subjected to survey to provide an up-to-date map of geographical distribution and level of risk of schistosomiasis and STH in the country. The sample size was determined according to the WHO guide (50 children per school) [21], but was increased to 100 per school considering the fewer sites used in this survey and the low prevalence data recorded previously within the country [7]. The survey sites (schools) were selected according to administrative districts (four schools per district) using a two-staged random sampling method to avoid two schools being selected from the same chiefdom to ensure a relatively even geographical coverage throughout the country. Therefore, in each district a list of chiefdoms was used as the sampling frame and four chiefdoms were first randomly selected. Within each selected chiefdom, a list of all primary schools was used as the sampling frame and one primary school was randomly selected. In total, 53 schools were selected for survey throughout the country. In Tonkolili which is ecologically heterogeneous with high altitude in the east and low altitude in the west, relevant chiefdoms were selected to represent the two ecological zones. Within each selected school, systematic sampling of children started from high grade classes and proceeded down through the grades with a view of balancing for sex. Approximately around 100 children aged 5 to 16 years per school (range: 36–134) were examined [22], [23]. In a few schools, due to large numbers of children present in the classes selected, up to 134 children were examined, while in one school, only 36 children were examined due to the small size of the school.

### Data collection

Following community sensitization and selection of school children, each pupil was supplied with a bottle for stool sample collection and an identification number was assigned. One stool sample from each selected pupil was collected. After collection of stool samples these were processed immediately and one slide per sample was prepared and examined in the field laboratory by the Kato-Katz thick smear technique using a 41.7mg template [24]. *S. mansoni* and STH infections were identified under light microscopes by experienced examiners. The survey received technical supervision from the WHO and the University of Sierra Leone.

Survey results were entered into Microsoft Excel. Prevalence of each parasite infection and corresponding differences between ages and sex was estimated taking into account the clustered design of the sampling, using the chiefdom as a primary sampling unit and including adjustments for the probability of sampling and finite population corrections for sampling without replacement in the Stata/SE 10.0 statistical package (StataCorp, College Station, Texas, USA).

**Figure 1:**
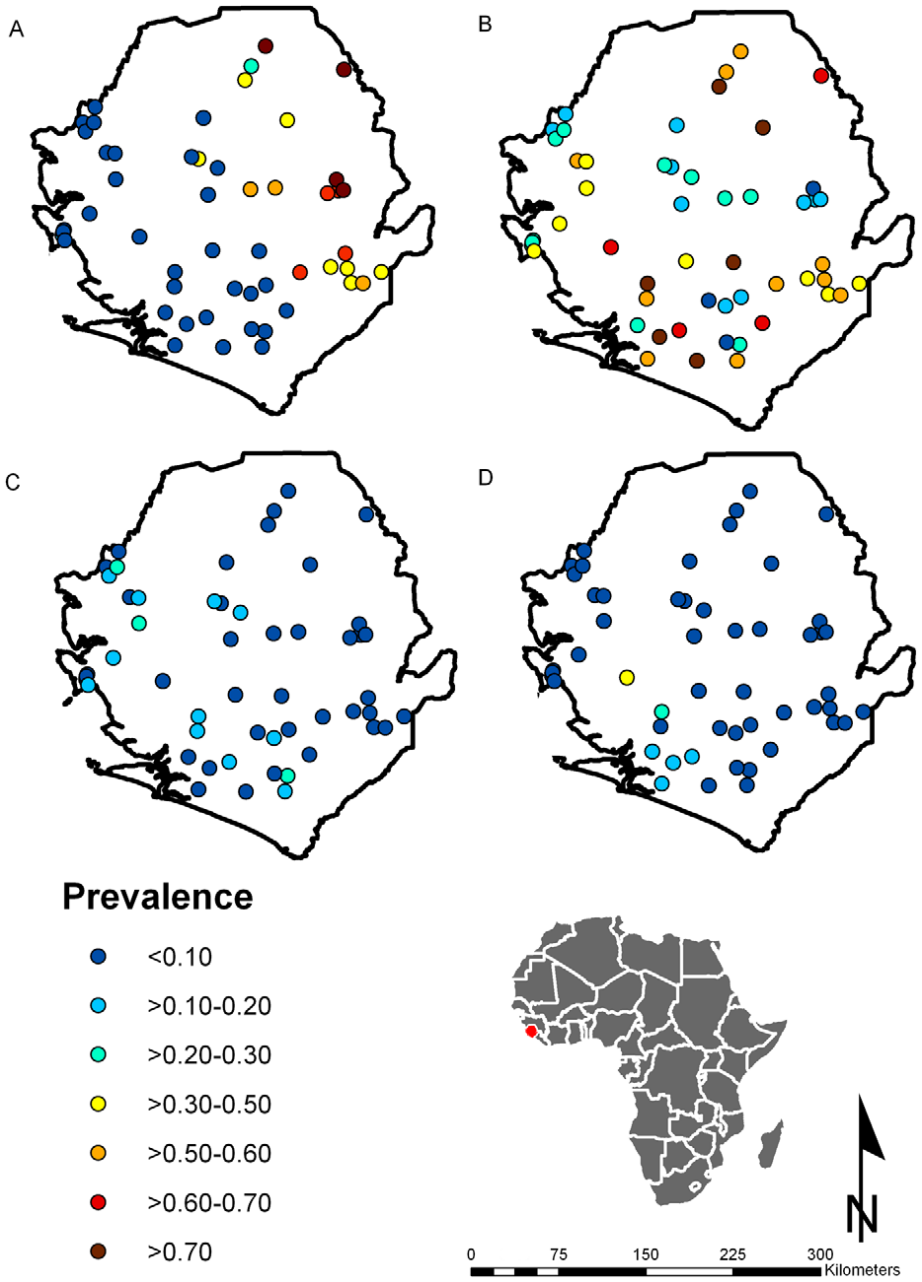
Geographical distribution of intestinal schistosomiasis and soil-transmitted helminthiasis in Sierra Leone. Observed point prevalence is shown by locations of the selected schools: (A) intestinal schistosomiasis, (B) hookworm, (C) *Ascaris lumbricoides*, and (D) *Trichuris trichiura*.

The coordinates of each sample site were recorded using hand-held units of global positioning system (GPS).The survey data were summarized as prevalence of infection by survey location. These summary data were plotted in a geographical information system (GIS) (ArcView version 9.3, ESRI, Redlands, CA) (Figure 1). Electronic data for land surface temperature (LST) and normalised difference vegetation index (NDVI) for a 5 km×5 km grid cell resolution were obtained from the National Oceanographic and Atmospheric Administration's (NOAA) Advanced Very High Radiometer (AVHRR; see Hay et al. [25] for details on these datasets) and the location of large perennial inland water bodies was obtained from the Food and Agriculture Organization of the United Nations (http://www.fao.org/geonetwork/srv/en/main.home) and the distance to the nearest perennial water body (DPWB) was extracted for each survey location in the GIS. A 5km resolution population surface derived from the Global Rural-Urban Mapping Project (GRUMP) beta product was obtained from the Center for International Earth Science Information Network (CIESIN) of the Earth Institute at Columbia University (http://sedac.ciesin.columbia.edu/gpw/global.jsp). Elevation data with a 5 km×5 km grid resolution, generated by a digital elevation model (DEM) from the Shuttle Radar Topography Mission (SRTM), were obtained from the Global Land Cover Facility (http://glcf.umiacs.umd.edu/index.shtml). All environmental datasets were linked to survey locations and values at each survey location were extracted in the GIS.

### Spatial risk prediction

The initial candidate set of predictor variables included population density, NDVI, LST, DPWB and elevation. Fixed-effects binomial logistic regression models of prevalence of infection for each parasite were developed in a frequentist statistical software package (Stata version 10.1, Stata Corporation, College Station, TX). In the preliminary multivariable models, elevation was not found to be significantly associated with hookworm infection risk. Population density, DPWB and LST were not found to be associated with *A. lumbricoides* infection; these variable were excluded from further analysis in the respective models (Wald's *P*>0.2). A quadratic association between LST and prevalence of infection was assessed for all models and was not found to improve model fit using the Akaike's Information Criterion.

We developed model-based geostatistical spatial prediction models [26] using the Bayesian statistical software, WinBUGS version 1.4 (Medical Research Council Biostatistics Unit, Cambridge, United Kingdom and Imperial College London, London, United Kingdom). All models had the covariates plus a geostatistical random effect, in which spatial autocorrelation between locations was modeled using an exponentially decaying autocorrelation function. Statistical notation of Bayesian geostatistical models, spatial interpolation and model validation procedures are presented in an additional file (Text S1).

### Determination of PCT strategies and calculation of target populations

After analysis of the data from each school and according to the geographical distribution (observed prevalence range) of the diseases within the country, endemic areas were categorized as high, moderate or low risk areas according to the prevalence thresholds (for details, see the WHO PCT guidelines [19]). As the PCT implementation was to be organized at each district level, the at-risk population was estimated for each district according to the endemicity category in the district and the projected population for 2009 from the 2004 National Census [20]. All the rural populations of the districts identified as having schistosomiasis in all survey sites are considered to be at-risk as they have occupations involving contact with infested water: fishermen, farmers, irrigation workers, artesian miners, women doing their domestic tasks and bathing. For PCT strategies, all school age children in endemic communities as well as those adults considered at high risk of infection were regarded as target population and to be treated according to the WHO PCT guidelines [19], [27].

## Results

### Prevalence of the diseases

A total of 5691 school children were selected and examined throughout the country. Among the total, 5069 school children had valid age, gender and parasitological data entries. There were 2563 males (50.6%) and 2506 females (49.4%). The mean age (± standard deviation) of pupils studied was 8.34±2.80 years (male 8.36±2.80 years and female 8.32±2.81 years). There was no significant difference in mean age between gender (p>0.05).

The overall point prevalence of *S. mansoni* infection in 5–16 year-old school children within the country was 18.4% (95% confidence interval (CI): 14.8–22.1%). There was no significant difference in overall prevalence between boys (18.5%, 95% CI: 14.9–22.2%) and girls (18.4%, 95% CI: 14.4–22.3%) (p>0.05). However, there is a significant difference between ages (p<0.001). In general, prevalence in children of 8–16 years old (25.1%, 95% CI: 20.1–30.1%, n = 2777) was significantly higher than that in 5–7 years old (10.4%, 95% CI: 8.1–12.7%, n = 2292) (p<0.001).

The overall point prevalence of hookworm infection in 5–16 year-old school children was 32.5% (95% CI: 28.7–36.3%) within the country. The overall prevalence was significantly higher in boys (34.6%, 95% CI: 30.7–38.5%) than in girls (30.4%, 95% CI: 26.4–34.3%) (p<0.001). Prevalence of hookworm in children of 8–16 years old (38.5%, 95% CI: 34.1–43.0%, n = 2777) was significantly higher than that in 5–7 years old (25.2%, 95% CI: 21.0–29.5%, n = 2292) (p<0.001). Low levels of infection were recorded for *A. lumbricoides* with an overall prevalence of 7.2% (95% CI: 5.8–8.6%). There was also overall low prevalence of *T. trichiura* (3.3%, 95% CI: 2.5–4.2%). *A. lumbricoides* and *T. trichiura* infections were not analyzed in detail. Overall prevalence of any STHs was 39.1% (95% CI: 37.8–40.5%) in 5–16 year-old school children within the country.

### Geographical distribution

To represent the level of endemicity of intestinal schistosomiasis and STHs, according to the above analysis, the prevalence in 8–16 years in each school was used in the following analysis to illustrate the geographical distribution of these NTDs within the country. The average number of 8–16 years old examined per school was 52 (range 28–87).

#### *Schistosoma mansoni*

Table 1 summarizes the results of *S. mansoni* prevalence in each district. Generally speaking, *S. mansoni* exists across the country with an uneven geographical distribution with a median prevalence (minimum-maximum observed range) of 6.0% (range 0–82.1%) in all schools surveyed (Figure 1A). The northeast half of the country is more heavily affected, such as Kono (range 63.8–78.3%), Koinadugu (range 21.6–82.1%) and Kailahun (range 43.5–52.6%), and part of Kenema (range 6.1–68.9%), Tonkolili (range 0–57.3%) and Bombali (range 2.1–42.6%) (Table 1). One school (Mabonto) in Tonkolili districts did not have a valid coordinate recorded and was not included in the prevalence maps (schistosomiasis prevalence 37.5% and STH prevalence 47.8%). The western coastal half of the country showed no or low (<10%) infection except that one school in the Western Area, MacDonald, had unexpectedly high prevalence of *S. mansoni* infection (18.8%).

**Table 1:** *S mansoni* prevalence in 8–16 years old by district and estimated target population.

District	No of children examined(male∶ female)	Mean age ±SD	*S. mansoni* prevalence (%)Median (observed min-max range)	Target population at risk(>5yrs)
Bo	163 (77∶86)	9.96±2.06	0 (0–0)	0
Bombali**	215 (115∶100)	10.24±2.04	7.5 (2.1–42.6)	363,060
Bonthe	196 (93∶103)	10.17±2.07	0 (0–0)	0
Kailahun***	187 (94∶93)	9.97±1.93	46.7 (43.5–52.6)	338,302
Kambia*	223 (125∶98)	10.49±2.09	2.7 (0–6.0)	75,342
Kenema***	208 (106∶102)	10.38±2.36	52.8 (6.1–68.9)	482,762
Koinadugu***	272 (155∶117)	11.34±1.69	44.4 (21.6–82.1)	247,962
Kono***	244 (107∶134)	10.23±2.21	72.3 (63.8–78.3)	233,027
Moyamba*	217 (121∶96)	10.42±2.20	0.9 (0–2.3)	60,388
Port Loko*	173 (96∶77)	9.99±1.82	0 (0–7.0)	122,734
Pujehun*	198 (100∶98)	10.62±2.27	2.7 (0–6.9)	75,001
Tonkolili***	280 (117∶163)	10.43±2.22	46.3 (0–57.3)	312,740
Western Area**	201 (105∶96)	10.39±2.09	5.6 (2.3–18.8)	289,907
Total	2777 (1411∶1366)	10.39±2.12	6.0 (0–82.1)	2,601,225

***High-risk districts: school age children and adults at high-risk in high-risk communities receive treatment annually.

**Moderate-risk districts: school age children and adults at-risk in moderate-risk communities receive treatment biennially.

*Low-risk districts: school age children in endemic communities may receive treatment twice during primary school.

#### Soil-transmitted helminthiasis

Table 2 summarizes the results of STHs in each district. STHs are distributed across the country, with high prevalence of hookworm infections, particularly in the western coastal areas and the northeast district Koinadugu (Figure 1B), while *A. lumbricoides* (Figure 1C) and *T. trichiura* (Figure 1D) are more evenly distributed with relatively low prevalence. In all schools surveyed across the country, there was a median prevalence (minimum-maximum observed range) of 48.3% (range 5.4–96.3%) for any STHs, in which hookworm infection has a median prevalence of 38.5% (range 5.4–95.1%), ascariasis 6.6% (range 0.0–25.0%), and trichuriasis 1.8% (range 0.0–30.2%).

**Table 2:** Prevalence of individual and overall STHs in 8–16 years old by district and estimated target population.

District	No of children examined	*A. lubricoides* prevalence (%)Median (observed min-max range)	*T. trichiura* prevalence (%)Median (observed min-max range)	Hookworm prevalence (%)Median (observed min-max range)	Any STHs prevalence (%)Median (observed min-max range)	Target population(>1yrs)
Bo	163	2.0 (0–11.8)	1.11 (0–3.6)	12.9 (5.4–71.4)	18.4 (5.4–71.4)	565,619
Bombali	215	8.6 (6.0–12.5)	0 (0–1.2)	17.9 (13.3–29.7)	25.2 (18.1–40.5)	421,407
Bonthe	196	1.0 (0–19.0)	13.7 (10.3–18.2)	56.5 (28.1–70.9)	62.7 (28.1–80.0)	143,770
Kailahun	187	0 (0-0)	0 (0–2.2)	49.8 (32.6–52.6)	49.8 (34.8–52.6)	398,842
Kambia	223	13.6 (8.2–21.3)	0 (0–6.7)	22.5 (16.3–26.7)	35.8 (24.5–48.3)	292,326
Kenema	208	0 (0–6.1)	1.8 (0–3.3)	53.3 (41.5–66.7)	53.3 (41.5–69.7)	561,123
Koinadugu	272	3.0 (0–8.6)	0 (0–3.0)	64.8 (53.7–95.1)	68.5 (56.7–96.3)	286,985
Kono	244	4.8 (3.2–6.9)	0 (0–1.6)	13.7 (8.1–15.5)	18.5 (11.3–20.7)	271,444
Moyamba	217	9.8 (6.6–12.7)	15.0 (3.4–30.2)	60.7 (42.6–70.9)	72.3 (45.9–80)	234,306
Port Loko	173	11.0 (1.7–25)	0.9 (0–3.1)	46.4 (38.5–51.7)	53.3 (50–56.1)	476,209
Pujehun	198	13.4 (5.1–21.8)	3.5 (0–5.5)	42.7 (8.5–75.9)	53.6 (15.3–75.9)	291,002
Tonkolili	280	7.0 (6.7–14.6)	2.2 (1.3–10)	24.7 (17.1–35.4)	33.3 (30–50)	371,819
Western Area	201	9.3 (4.5–13.2)	5.6 (3.6–8.4)	28.5 (25–44.7)	41.7 (31.8–60.5)	1,124,840
**Total**	2777	**6.6 (0–25)**	**1.8 (0–30.2)**	**38.5 (5.4–95.1)**	**48.3 (5.4–96.3 )**	**5,439,692**

### Spatial risk prediction

Model results (Table 3) indicated that: prevalence of *S. mansoni* was positively associated with population density and elevation; prevalence of hookworm infection was negatively associated with population density and LST; prevalence of *A. lumbricoides* was negatively associated with elevation; and prevalence of *T. trichiura* was negatively associated with LST and elevation and positively associated with DPWB. Phi (*ϕ*) indicates the rate of spatial decay of spatial autocorrelation and varied from 11.98 and 4.72 for *T. trichiura* and *A. lumbricoides*. This indicates that, after accounting for the effect of covariates, the radii of the clusters were approximately 70km in case of *A. lumbricoides* and 28km in case of *T. trichiura* (note, *ϕ* is measured in decimal degrees and 3/*ϕ* determines the cluster size; one decimal degree is approximately 111 km at the equator). The tendency for spatial clustering was the strongest for *S. mansoni* and the weakest for *T. trichiura* (the higher value the spatial variance parameter the higher the tendency for spatial clustering).

**Table 3:** Spatial effects for prevalence of *S. mansoni*, hookworm, *A. lumbricoides* and *T. trichiura* in schoolchildren in Sierra Leone.

Variable	*S. mansoni*	Hookworm	*A. lumbricoides*	*T. trichiura*
Population density*	0.56 (0.22,0.99)	−0.27 (−0.52,−0.01)		−0.28 (−0.81,0.22)
DPWB*	−0.27 (−1.02,1.45)	0.45 (−0.33,1.11)		0.95 (0.09,1.77)
NDVI*	0.21 (−0.48,0.86)	0.19 (−0.15,0.52)	−0.44 (−0.83,−0.06)	0.16 (−0.30,0.59)
LST*	0.65 (−0.50,1.79)	−0.62 (−1.26,−0.04)		−1.30 (−2.11,−0.60)
Elevation*	1.03 (0.09,2.03)		−0.42 (−1.40,0.38)	−0.80 (−1.56,−0.06)
Intercept	−1.97 (−3.34,2.37)	−0.35 (−0.92,0.68)	−2.99 (−3.93,−2.21)	−3.99 (−4.54,−3.50)
*ϕ* (rate of decay of spatial correlation)	6.12 (0.48,17.86)	6.37 (1.24,16.66)	4.72 (0.57,15.87)	11.98 (2.94,19.58)
*σ* ^2^ (variance of spatial random effect)	4.18 (1.20,17.18)	1.22 (0.59,2.91)	1.46 (0.38,4.99)	0.80 (0.14,2.07)

*Variables were standardised to have mean = 0 and standard deviation = 1; Abbreviations: NDVI - Normalised difference vegetation index; LST- Land surface temperature; DPWB - Distance to nearest perennial water body.

**Figure 2:**
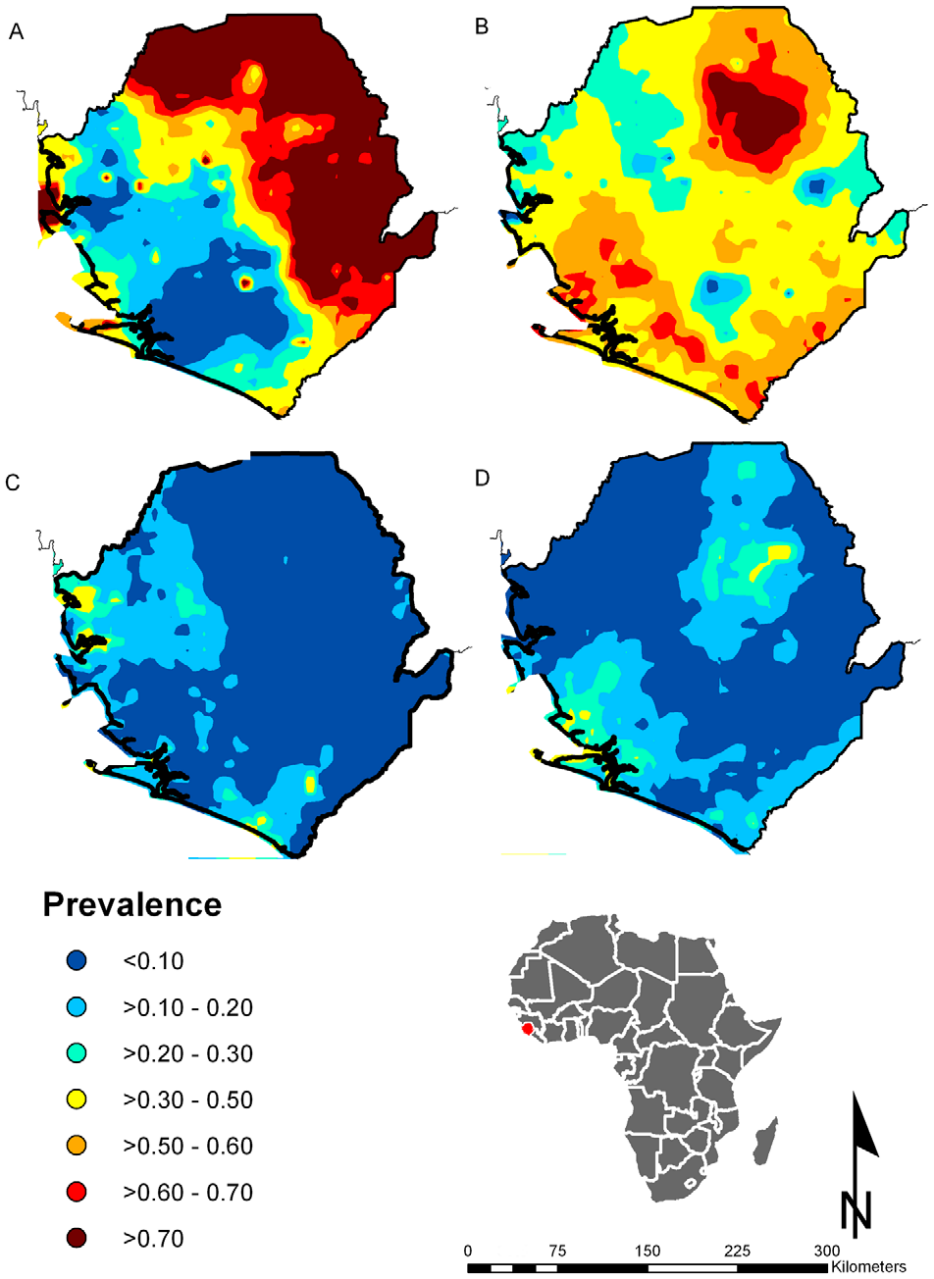
Predicted spatial distribution of intestinal schistosomiasis and soil-transmitted helminthiasis in Sierra Leone. (A) intestinal schistosomiasis, (B) hookworm, (C) *Ascaris lumbricoides*, and (D) *Trichuris trichiura*.

We found a large cluster of high risk of *S. mansoni* infection (prevalence >70%) in a region covering the north and most of the eastern areas of the country (Figure 2A). The predicted prevalence of hookworm infections was high across Sierra Leone with a large cluster of high infection risk (prevalence >70%) in the north-eastern part of the country (Figure 2B). The risk of *A. lumbricoides* (Figure 2C) and *T. trichiura* (Figure 2D) was predicted to be highest (prevalence >20%) in western, central and southern areas of the country. All models showed acceptable predictive ability (i.e. AUC>70%).

### Estimation of at-risk populations for schistosomiasis and STH

According to the current *S. mansoni* prevalence distribution and the WHO PCT guidelines [19] which is now widely implemented in several countries in sub-Saharan Africa [27], [28], communities in the high-risk endemic areas in Koinadugu, Kono, Kailahun and part of Kenema and Tonkolili are justified for annual PCT for schistosomiasis in school age children and adults at high risk with an estimated target population of around 1.5 million (Table 1). About 730,000 people are estimated in the moderate-risk endemic areas in Bombali, Kenema and Tonkolili, where treatment for school age children and at-risk adults once every two years is justified. A further 330,000 school age children are in the low-risk endemic areas, who may require treatment twice during their primary education.

According to the STH prevalence maps, the entire population in the country is at risk of STH. In particular, those in five coastal districts, Koinadugu and part of Bo, about 2.84 million people, are at high risk of STH infection (Table 2), justified for PCT twice a year to pre-school children, school age children and adults at high risk. Another 2.6 million people in the remaining areas are at moderate risk of STH infection, therefore justified for annual treatment to pre-school children, school age children and at-risk adults. A total target population was 5.44 million.

## Discussion

Integrated control of NTDs according to the WHO PCT guidelines requires careful planning [22]; therefore, it is essential to map the distribution of each disease in order to determine the disease-specific PCT strategy in each community in the country. The results presented here are the first of such national survey of schistosomiasis and STH in Sierra Leone, which provided a foundation for planning the integrated drug delivery in the national integrated control program on NTDs in the country. The results from schools surveyed throughout the country suggest that school age children in Sierra Leone suffer from high level of *S. mansoni* infection with estimated prevalence of up to over 82%, and level of infection with STH is even higher with estimated prevalence of up to 96%. It is noted that only one Kato-Katz slide was used for diagnosis during this survey and those children with light infections could have been missed. Therefore, the actual level of infection may have been higher than observed. It is likely that in many places infection with STH and in some places infection with *S. mansoni* may have been universal. The results highlighted the need for control of these NTDs in the country. Due to the emphasis of the survey on prevalence maps and limited human resources within the national team, the number of parasite eggs per slide was not properly recorded, and therefore intensity of infection was not analyzed in this paper. Due to limited data available on *S.haematobium*, distribution of urinary schistosomiasis is not included in this paper. Further survey on *S.haematobium* has been carried out and it will be described separately.

The results were broadly in line with and confirmed the previous findings that *S. mansoni* infections and STH were widespread in Sierra Leone with *S. mansoni* infections more heavily distributed in the Northeast areas and STH evenly distributed across the country [6], [7], [8], [9], [10], [11]. Geographical distribution of *S. mansoni* depends on many environmental factors [29]. Spatial analysis taking into consideration of population density, NDVI, LST, DPWB and elevation suggests that *S. mansoni* was positively associated with population density and elevation. A good example is Tonkolili district. It lies in the center of Sierra Leone. To the east, at high altitude it borders with Kono district and the 2 chiefdoms Konike-Sanda and Kunike-Barina on the Kono border had high prevalence for *S. mansoni* 57.3% and 55.2% respectively in the 8–16 years old. In the west of Tonkolili at low altitude Kolifa-Rowala no infection with *S. mansoni* was detected in the 8–16 years old. In general the results follow the expectations of prevalence at altitude greater than 250 meters above sea level [7]. Spatial analysis also suggests that *S. mansoni* has the strongest tendency for spatial clustering. Hookworm is widely distributed across the country but with high prevalence in the western coastal areas and northeast district Koinadugu, which is not related to elevation but negatively associated with population density and LST. *A. lumbricoides* and *T. trichiura* are more evenly distributed in the country though the prevalence was relatively low. The geographical distribution of STHs in the country looks different from those found in other countries [30], [31]. There may be indeed a difference in STH distribution between West and East Africa [23].

The survey provided important tools for the national NTD control program to plan the implementation strategies for each district and for each disease. Multilevel sampling is well accepted sampling method for such surveys; however, in the current survey design, relatively few survey sites with a large sample size for each site were randomly selected, which may have not given an accurate prediction of geographical distribution of the diseases, in particular, schistosomiasis throughout the country. As in Bo district, the *S. mansoni* prevalence in this survey was significantly lower than senior local clinical opinion and results reported by other investigators [7]. It was thought that the chiefdoms with high prevalence of *S. mansoni* had consequently been missed due to the random sampling method used and a relatively few sites surveyed. It would have given a better estimate if more survey sites with a relatively smaller sample size for each site had been used, together with considering the ecological nature of the diseases, particularly schistosomiasis which is a focal disease. Further survey would be needed to validate the spatial distribution of the diseases in the country.

An unexpected high prevalence of *S. mansoni* was found in MacDonald village, Waterloo, Western Area. It was initially attributed to the presence there of many internally displaced persons as a result of the civil war during 1991–2002. A camp for the internally displaced population had accommodated many thousand people nearby and many had settled in the Western Area since the camp was disbanded by the UN. This finding warrants further investigation to ascertain if the children infected had previously lived in the North or East or were in fact indigenous to the Western Area. The classification of the transmission risk for the persons living in the Western Area is currently being further investigated.

Remarkably low levels of prevalence were recorded for *A. lumbricoides* (7.2%) and *T. trichiura* (3.9%) in the 8–16 years old. Numerous publications in the 1990s recorded rural prevalence in school age children of 32–93% for *A. lumbricoides* and 1–81% for *T. trichiura* [8], [9], [10], [13], [14]. This remarkable reduction in prevalence may be partly explained by programs de-worming school-going children performed by a non-governmental organization (St Andrews Clinics for Children-Sierra Leone) and UN agencies since 2004 and the preventative chemotherapy for onchocerciasis and lymphatic filariasis which started distribution of ivermectin in 2005 and ivermectin plus albendazole in 2007 [32].

### Implementation strategies of PCT for schistosomiasis

Based on the results of this survey, PCT with praziquantel for schistosomiasis was performed for the first time in Sierra Leone in June 2009 and was focused in the northeast districts. As this was the first round of such large scale treatment, significant side effects in heavily parasitized children were anticipated: headaches, stomach aches, nausea, vomiting and diarrhea. To ease this concern, PCT was phased in, and only school-going children in these districts (including district cities) were treated in the first round (a total of 562,980). Rural adult populations of the districts identified as having a high risk of *S. mansoni* infection are to be targeted in 2010. All school age children in the high prevalence districts, Kono and Koinadugu, will be targeted again in the second round in 2010.

### Implementation strategies of PCT for STHs

STH distribution is widespread in the country, particularly with high prevalence of hookworm infection (Figures 1B and 2B). A government report suggests that there was a high prevalence of anemia in children in the country [33]. Considering these two factors, it was decided that 12 out of 13 districts require PCT twice a year at the beginning of the control program. The decision took into consideration the prevalence range observed in each district and the likely underestimation of one Kato-Katz slide used in the survey on the actual prevalence. The existing de-worming programs for school age children in the country funded and organized by UN agencies and NGOs have now been fully coordinated and integrated under the National School and Adolescent Health program. The LF elimination commenced with ivermectin and albendazole distribution for all persons over 5 years of age in 2007, scaling up to national coverage in 2010, including the capital Freetown and other cities. This provides the first round of PCT for STHs. The second round of PCT with mebendazole for STHs in 2009 was included in PCT for schistosmiasis in the districts receiving praziquantel, 6 months after PCT-LF. The National School and Adolescent Health program plans to scale up to national coverage of the second round of PCT for school age children and to integrate this activity into the Mother and Child Health Weeks. The MoHS has also included the provision of anthelminthic to pregnant women at the first visit in the second trimester into their basic ante-natal care package and district health indicators. It is noted that given the predominant hookworm infection among STHs it would be desirable to use albendazole as the drug of choice in de-worming in the country [34].

In conclusion, the first national survey on distribution of intestinal schistosomiasis and STH in Sierra Leone was carried out in 2008. The results showed that school age children in Sierra Leone suffer from high prevalence of STH throughout the country and high prevalence of *S. mansoni* infection in the northeast half of the country, highlighting the need of control on these NTDs. It provided a platform for the Ministry of Health and Sanitation to plan the implementation strategies in the national NTD control program. The first round of pilot PCT campaign in school children was conducted in the moderate and heavy endemic areas in 2009 and the plan is made for the second round of treatment to expand the PCT coverage to a larger population including adults at high risk in the northeast areas.

## Supporting Information

Checklist S1 STROBE checklist.
(0.08 MB DOC)

Text S1 Supplementary technical information.
(0.04 MB DOC)

## References

[1] Hotez PJ, Kamath A (2009). Neglected tropical diseases in sub-saharan Africa: review of their prevalence, distribution, and disease burden.. PLoS Negl Trop Dis.

[2] Hotez PJ, Molyneux DH, Fenwick A, Kumaresan J, Sachs SE (2007). Control of neglected tropical diseases.. N Engl J Med.

[3] Hotez PJ, Brindley PJ, Bethony JM, King CH, Pearce EJ (2008). Helminth infections: the great neglected tropical diseases.. J Clin Invest.

[4] Liese B, Rosenberg M, Schratz A (2010). Programmes, partnerships, and governance for elimination and control of neglected tropical diseases.. Lancet.

[5] Stothard JR, Chitsulo L, Kristensen TK, Utzinger J (2009). Control of schistosomiasis in sub-Saharan Africa: progress made, new opportunities and remaining challenges.. Parasitology.

[6] Gbakima AA, Sahr F (1995). Intestinal parasitic infections among rural farming communities in eastern Sierra Leone.. Afr J Med Med Sci.

[7] World Health Organization (2010). Atlas of global distribution of schistosomiasis.. http://www.who.int/wormcontrol/documents/maps/country/en/.

[8] Webster J, Hodges M, Crompton DWT, Walters DE (1990). Intestinal parasitic infections in children from Freetown, Sierra Leone.. J Sierra Leone Med Dental Association.

[9] Bayoh M, Hodges M (1993). Effect of levamisole chemotherapy on geohelminthiases Sierra Leone.. J Sierra Leone Med Dental Association.

[10] Williams RA, Koroma MM, Hodges M (1997). Comparison of albendazole and levamisole chemotherapy on prevalence and intensity of common soil-transmitted helminth infections in school children, Sierra Leone.. West Afr J Med.

[11] Gbakima AA, Sherpard M, White PT (1994). Intestinal helminth infections in rural school children in Njala, Sierra Leone.. East Afr Med J.

[12] Bayoh M, Hodges M, Crompton DWT (1992). Identification of *Necator americanus* from a community in Bo, Sierra Leone.. J Sierra Leone Med Dental Association.

[13] Williams RAM, Hodges M (1995). The Effects of levamisole chemotherapy on the growth of primary school children and the prevalence and intensity of common STHs in Sierra Leone.. J Sierra Leone Med Dental Association.

[14] Koroma MM, Williams RA, de la Haye RR, Hodges M (1996). Effects of albendazole on growth of primary school children and the prevalence and intensity of soil-transmitted helminths in Sierra Leone.. J Trop Pediatr.

[15] Stephenson LS, Latham MC, Kurz KM, Miller D, Kinoti SN (1985). Urinary iron loss and physical fitness of Kenyan children with urinary schistosomiasis.. Am J Trop Med Hyg.

[16] Friedman JF, Kanzaria HK, Acosta LP, Langdon GC, Manalo DL (2005). Relationship between *Schistosoma japonicum* and nutritional status among children and young adults in Leyte, the Philippines.. Am J Trop Med Hyg.

[17] Olson CL, Acosta LP, Hochberg NS, Olveda RM, Jiz M (2009). Anemia of inflammation is related to cognitive impairment among children in Leyte, the Philippines.. PLoS Negl Trop Dis.

[18] Stephenson LS, Latham MC, Ottesen EA (2000). Malnutrition and parasitic helminth infections.. Parasitology.

[19] World Health Organization (2006). Preventive chemotherapy in human helminthiasis: coordinated use of anthelminthic drugs in control interventions.

[20] Koroma DS, Turay AB, Moigua MB (2006). Republic of Sierra Leone, 2004 population and housing census.

[21] Montresor A, Crompton DWT, Hall A, D.A.P. B, Savioli L (1998).

[22] Brooker S, Kabatereine NB, Smith JL, Mupfasoni D, Mwanje MT (2009). An updated atlas of human helminth infections: the example of East Africa.. Int J Health Geogr.

[23] Clements AC, Deville MA, Ndayishimiye O, Brooker S, Fenwick A (2010). Spatial co-distribution of neglected tropical diseases in the east African great lakes region: revisiting the justification for integrated control.. Trop Med Int Health.

[24] Katz N, Chaves A, Pellegrino J (1972). A simple device for quantitative stool thick-smear technique in schistosomiasis mansoni.. Rev Inst Med Trop Sao Paulo.

[25] Hay SI, Tatem AJ, Graham AJ, Goetz SJ, Rogers DJ (2006). Global environmental data for mapping infectious disease distribution.. Adv Parasitol.

[26] Diggle PJ, Moyeed RA, Tawn JA (1998). Model-based geostatistics.. Applied Statistics.

[27] Zhang Y, Koukounari A, Kabatereine N, Fleming F, Kazibwe F (2007). Parasitological impact of 2-year preventive chemotherapy on schistosomiasis and soil-transmitted helminthiasis in Uganda.. BMC Med.

[28] Kabatereine NB, Brooker S, Koukounari A, Kazibwe F, Tukahebwa EM (2007). Impact of a national helminth control programme on infection and morbidity in Ugandan schoolchildren.. Bull World Health Organ.

[29] Kabatereine NB, Brooker S, Tukahebwa EM, Kazibwe F, Onapa AW (2004). Epidemiology and geography of *Schistosoma mansoni* in Uganda: implications for planning control.. Trop Med Int Health.

[30] Brooker S, Kabatereine NB, Tukahebwa EM, Kazibwe F (2004). Spatial analysis of the distribution of intestinal nematode infections in Uganda.. Epidemiol Infect.

[31] Kabatereine NB, Tukahebwa EM, Kazibwe F, Twa-Twa JM, Barenzi JF (2005). Short communication: soil-transmitted helminthiasis in Uganda: epidemiology and cost of control.. Trop Med Int Health.

[32] Ottesen EA, Hooper PJ, Bradley M, Biswas G (2008). The global programme to eliminate lymphatic filariasis: health impact after 8 years.. PLoS Negl Trop Dis.

[33] MinistryHSSL (2008). Sierra Leone demographic and health survey, preliminary report.

[34] Keiser J, Utzinger J (2008). Efficacy of current drugs against soil-transmitted helminth infections: systematic review and meta-analysis.. JAMA.

